# Multiview Deep Forest for Overall Survival Prediction in Cancer

**DOI:** 10.1155/2023/7931321

**Published:** 2023-01-18

**Authors:** Qiucen Li, Zedong Du, Zhikui Chen, Xiaodi Huang, Qiu Li

**Affiliations:** ^1^Dalian University of Technology, School of Software, China; ^2^Chengdu Second People's Hospital, Department of Oncology, China; ^3^Charles Sturt University, School of Computing, Mathematics and Engineering, Australia; ^4^West China Hospital, Department of Oncology, China

## Abstract

Overall survival (OS) in cancer is crucial for cancer treatment. Many machine learning methods have been applied to predict OS, but there are still the challenges of dealing with multiview data and overfitting. To overcome these problems, we propose a multiview deep forest (MVDF) in this paper. MVDF can learn the features of each view and fuse them with integrated learning and multiple kernel learning. Then, a gradient boost forest based on the information bottleneck theory is proposed to reduce redundant information and avoid overfitting. In addition, a pruning strategy for a cascaded forest is used to limit the impact of outlier data. Comprehensive experiments have been carried out on a data set from West China Hospital of Sichuan University and two public data sets. Results have demonstrated that our method outperforms the compared methods in predicting overall survival.

## 1. Introduction

Overall survival (OS) refers to the length of a living period from the start of treatment for a patient diagnosed with the disease who is still alive [1]. It is the gold standard primary endpoint for evaluating the outcome of any drug, biologic intervention, or procedure that is assessed in oncologic clinical trials [2]. The prediction of OS provides a useful reference for doctors and patients to choose treatment methods. In particular, a reliable OS prediction result can help doctors to choose perioperative adjuvant therapy and frequency of surveillance and make patients more aware of their disease and have more confidence in doctors. OS prediction in cancer has aroused many researchers' interest.

At present, accurate OS prediction is still a steep challenge. On the one hand, the multiview data with high dimensions require the model to mine relevance and reduce redundancy between different views [3]. On the other hand, clinical application requires interpretability, which limits deep learning from getting widely recognized [4].

OS prediction relies on typical multiview data. The data are collected from diverse domains or obtained from various feature extractors and exhibit heterogeneous properties [5]. As we know, patient basic information, pathological diagnosis, and physiological indicators all affect the prediction results. Variables of each data record can be naturally partitioned into groups [6]. Each group can be regarded as a view. Many machine learning algorithms [7–9] have been applied to an OS prediction task. However, it is difficult for a single model to comprehensively use all multiview data [10]. Different models are needed for different views to capture the deep, latent relationships between these data and survival time. In addition, the redundancy between data from different views is not insignificant [11]. If we put all clinical information together, this may result in overfitting. Thus, the multiview data with both relevance and redundancy pose a great challenge for building a good machine learning model.

Due to the excellent performance of deep networks in mining multiview data, deep learning has been applied to the prognosis of cancer [12–14]. However, a deep learning model is a black box. The parameters obtained from model training cannot be explained by medical knowledge or clinical experience. For addressing this problem, a deep learning architecture called multigrained cascade forest (gcForest) [15] builds an effective, explainable model by integrating random forests. The parameters in the random forest composed of multiple decision trees can correspond to detection indicators and explain how the model obtains the prediction results. In the experiment section, we also compare the parameters with the clinical guidelines to verify the interpretability of our model.

However, clinical data is usually inadequate compared to the large number of parameters in deep learning. Limited training data often leads to overfitting. Various strategies such as “early-stopping,” “network-reduction,” “data-expansion,” and “regularization” are commonly used to address the issue of overfitting [16]. In general, the main idea to prevent overfitting is to enhance generalization or augment the training data [17]. We also follow these two ideas to improve the model. Feature extraction can effectively reduce model complexity and improve generalization. Many feature selection methods have been applied to clinical data and achieved good results [18]. In multiview deep forest, we design a feature learning method based on the information bottleneck (IB) theory, which can reduce the data dimension and maintain mutual information between compressed features and labels [19]. Meanwhile, a resampling strategy is used for data augmentation.

In this paper, we propose a novel approach to predicting overall survival in cancer. In particular, we propose a multiple kernel learning method for multiview data. It is capable of obtaining and integrating features from each view. To avoid overfitting, the IB theory and resampling strategy are applied to improve the information density of clinical data. For dealing with the influence of noise in survival data, a pruning strategy is proposed to filter the decision trees at each level. It can reduce the disturbance of special cases to the model while keeping generalization. In general, multiview deep forest (MVDF) can improve the reliability of prediction and assist decision-making in disease prevention and management. The main contributions of this paper are summarized as follows:

- A multiple kernel learning method based on random forest is proposed to mine and integrate the features of multiview clinical data
- Based on the IB theory, we propose a feature learning method that reduces data dimension and augments training data to avoid overfitting
- Using a pruning strategy, we screen the decision trees of each level in the cascade forest to reduce the impact of low-quality data
- We have conducted comprehensive experiments by applying the deep forest approach to predicting overall survival in cancer, validating the performance of our model on clinical data

The rest of this paper is organized as follows.  Section 2 is the related work, followed by presenting our multiview deep forest.  Section 3 introduced the proposed multiview deep forest method.  Section 4 presents the experiments, and Section 5 concludes this paper.

## 2. Related Work

### 2.1. Overall Survival Prediction

As mentioned before, overall survival (OS) refers to the time which begins at diagnosis (or at the start of treatment) and up to the time of death. OS is usually used as an indication of how well a treatment for cancer works. The current methods for predicting OS in a clinic are based mainly on nomograms [20, 21]. It is a useful tool to predict the probability of clinical events via a straightforward and clear diagram. Except for using nomogram, these methods consider potential prognostic factors including age, sex, family history of cancer, tumor location, the size of tumor, grade of tumor, and the total number of lymph nodes examined and lymph node ratio [22]. Limited by the model structure, the nomogram, however, is difficult to learn the complex internal relationships between these indicators, such as the similarity of demographic characteristics between patients, the potential correlation between diagnosis and patients, and the correlation between patients and their physiological indicators. Therefore, this kind of method is often used for clinical rapid diagnosis, but not for long-term postoperative survival prediction with multiview data.

In the past decade, machine learning techniques, especially deep learning, have been popular among researchers in building cancer survival prediction models [12, 13, 23, 24]. Ahmad et al. [25] employed radix tree structure to decrease the computational difficulty in deep learning. Poonguzhali et al. [26] proposed an automated deep residual U-Net segmentation method for medicinal image database. Some researches have focused on the interpretability of deep learning model, especially for medical data. Wang et al. [27] introduce the physician's diagnostic process into the workflow, and Song et al. [28] introduce medical diagnostic knowledge. However, the hidden information learned by the deep models is still vague. Therefore, the current deep learning research is still mainly focused on medical image, instead of clinical data.

### 2.2. Deep Forest

The concept of the deep forest comes from the integration of random forests. Zhou and Feng proposed a decision tree ensemble method called gcForest (multigrained cascade forest) in 2017 [15]. In this cascade structure, each cascade is represented as an ensemble of decision tree forests. Each cascade is represented as an ensemble of decision trees. In addition, to adapt to different data types and obtain sufficient features from a small number of samples, gcForest also includes a multigranularity scanning structure. Cascade structure and scanning structure are independent of each other and can be used alone.

Many researchers have done further studies on this method [29, 30]. Katzmann et al. [31] added a neural network in the cascaded forest to learn the weights of different forests for superior performance on medical imaging data sets with only small data. Deep differentiable random forests connect split nodes to the top layer of convolutional neural networks (CNNs) and deal with inhomogeneous data by jointly learning input-dependent data partitions at the split nodes and distributions at the leaf nodes [32]. Zhang et al. [33] designed a lightweight indoor robot positioning system with deep fuzzy forest, which verifies that deep forest can be used for data calculation in IoT terminal devices. However, the existing methods still encounter many problems, such as low data quality, slow convergence, and overfitting. Furthermore, the sampling method of multigranular sampling window is not applicable because the indicators of patients have no temporal or spatial association. Instead, medical expert knowledge should be added to improve the accuracy of the model. Thus, they are not suitable for medical data and difficult to apply to the field of OS prediction.

### 2.3. Information Bottleneck Method

The information bottleneck (IB) method is a technique in information theory introduced by Tishby et al. [34]. It has been introduced to deep learning to compress data *X* into latent representation *Z* while maintaining mutual information with target prediction *Y*. This is achieved by relaxing the sufficiency condition to capture a fraction of *I*(*Z*; *Y*), the mutual information with *Y*. The algorithm minimizes the following functional with respect to conditional distribution *p*(*z*|*x*):min_*p*(*z*|*x*)_*I*(*Z*; *T*) − *βI*(*Z*; *Y*), where *β* is a Lagrange multiplier controlling the size of the information bottleneck.

Using the information bottleneck to eliminate irrelevant information can make the classifier more generalized and less overfitting [35, 36]. This is because the classifier can only extract relevant information without being misled by irrelevant information. For multiview data, the IB theory can be applied to filter out irrelevant and noisy information from multiple views and learn an accurate joint representation [37]. It performs well even in unsupervised multiview learning tasks [38]. However, few pieces of research explore the application of the IB theory to the decision tree model. We try to combine the IB theory with the gradient boosting decision tree to improve the performance of our model.

## 3. Multiview Deep Forest

**Figure 1:**
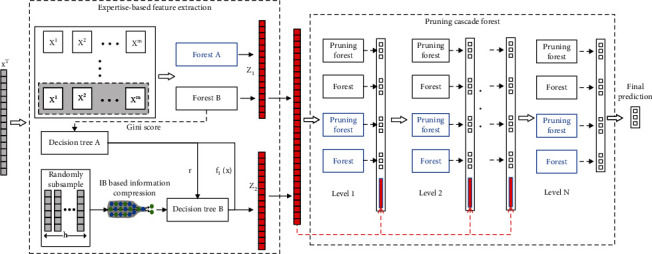
The overall procedure of multiview deep forest (MVDF). The total number of levels of cascaded forests is determined automatically.

Aiming at the multiview and low-quality clinical OS prediction data, we propose a multiview forest model, which mainly includes two feature learning methods and a cascade forest pruning strategy.  Figure 1 illustrates the overall procedure of MVDF.

### 3.1. Multiview Feature Extraction

Inspired by convolutional neural networks and recurrent neural networks, gcForest learns features of different views with a procedure of multigrained scanning. However, in the field of OS prediction, the temporal or spatial relationship between input data is not obvious. Instead, the logical relationships between different views of the data are essential. Therefore, we adopt a resampling strategy to make full use of the multiview data and propose a multiview feature extraction method.

**Figure 2:**
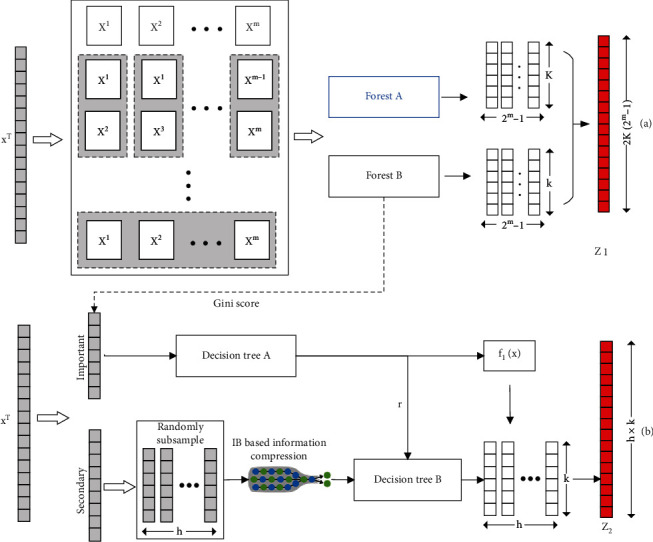
Multiview feature extraction. (a) Views are formed according to their relationship between original data. (b) Knowledge learned in random forests is used to select important indicators.

Suppose one data sample *x* has *n* indicators to describe the state of a patient. Firstly, we divide these indicators into *m* views according to the data acquisition method, data type, and description object. The whole data set can be represented by *X* = [*X*^1^, *X*^2^, ⋯, *X*^*m*^] as *m* view subsets. Taking the data from different views as the training data as shown in Figure 2(a), we can get different random forests, that is, view-proprietary feature extractors. Based on the idea of ensemble learning, *X*^1^, *X*^2^, ⋯, *X*^*m*^ is then combined as training data to obtain multiple weak feature extractors. Finally, multiview feature *z*_1_ is obtained by combining the outputs of all weak feature extractors. Since the output of a random forest is a class vector, the output of each weak feature extractor is a *K*-dimensional vector, where *K* is the number of classifications.

To enhance the diversity of ensemble construction, we used two different random forests. Forest A is a completely random tree forest, while forest B is a random forest. Each completely random tree forest contains several completely random trees, generated by randomly selecting a feature for splitting at each node of the tree and growing a tree until a pure leaf; i.e., each leaf node contains only the same class of instances. Similarly, each random forest contains several trees, by randomly selecting $\sqrt{n}$ number of features as candidates and choosing the one with the highest Gini value for a split. The number of trees in each forest is a hyperparameter, which is shown in Table 1.

The association relationship between different classes can be obtained by conducting the permutation and combination of *X*^*m*^. Therefore, for one sample, a total of 2^*m*^ − 1 three-dimensional class vectors can be obtained by a forest and produce a *K*(2^*m*^ − 1)-dimensional feature. In the prediction of OS after gastrectomy with our data set, *K* = 3, *m* = 5 and the five views are basic information of patients, such as height, weight, and cancer history of immediate relatives; pathological indexes, such as cancer classification, number of cancer nodules, and tumor diameter; treatment methods, such as chemotherapy, radiotherapy, and operation; preoperative tumor markers, such as AFP, hemoglobin, and CA125; and postoperative tumor markers. In other words, when *m* = 5, each original sample *x* is transformed into a 186-dimensional feature *z*_1_.

### 3.2. Feature Extraction Based on IB

The main purpose of this method is to prevent overfitting. The parameters of the decision tree are interpretable in that they are associated with semantic indicators. We can easily extract information from the trained model parameters as a priori knowledge for further learning. Some important clinical indicators of the original data can therefore be selected based on priori knowledge. We can get a class vector with important indicators and then adjust it with other secondary indicators as shown in Figure 2(b). However, for the use of boost ensemble learning, each classifier needs to meet the requirements of the low variance as much as possible, which is difficult for the secondary indicators with low information density and high dimension. Therefore, we apply the IB theory to construct a fully connected network to compress secondary indicators before training.

First, we analyze the trained random forests in multiview feature extraction and select important indicators. In this paper, we use the Gini score to measure the importance of each indicator. For the *i*-th indicators, *i* = 1, 2, ⋯, *n*, its Gini score VIM_*i*_ means the average change of the *i*-th indicator to the node purity in all random forests. All the nodes in the forests divided by this indicator form a set of *J*_*i*_. For any node *j* ∈ *J*_*i*_, the Gini score is calculated as

(1) $$\begin{array}{c} {\text{VIM}}_{i}^{j}={\text{GINI}}_{i}^{j}-{\text{GINI}}_{i}^{jl}-{\text{GINI}}_{i}^{jr}, \end{array}$$

where GINI_*i*_^*j*^ is the Gini value of the *i*-th indicator of node *j*, which is used to measure the information purity. For the *i*-th indicator, GINI_*i*_^*jl*^ is the Gini value of the left child node of node *j*, while GINI_*i*_^*jr*^ is the Gini value of the right child node. The Gini score of indicator *x*_*i*_ is the sum of Gini scores of all nodes in set *J*_*i*_ as VIM_*i*_ = ∑_*j*∈*J*_*i*__VIM_*i*_^*j*^. It should be noted that random forest A is a completely random tree forest, and the nodes in a completely random decision tree do not reflect the importance of indicators. Therefore, only random forest B is used in this step.

After calculating the Gini scores of all indicators, take the five indicators with the highest scores as important indicators. In addition to the knowledge learned from the data, the experience of doctors is also very valuable. In clinical rapid diagnosis, doctors usually make judgments based on a few indicators. Specifically, T stage, number of metastatic local lymph nodes, lymph node-positive rate, adjuvant chemotherapy, and diameter of tumour [22] are chosen as important features by doctors for the task of OS prediction after gastrectomy.

Then, data can be divided into *X*^*v*^ and *X*^*s*^. *X*^*v*^ is the data with important indicators obtained by Gini score, and doctor forms a set *X*^*v*^, and *X*^*s*^ = *X* − *X*^*v*^. In other words, *x*^*v*^ ∈ *X*^*v*^ and *x*^*s*^ ∈ *X*^*s*^ are different indicators describing the same patient. Since there are still many secondary indicators with less information, we compress the secondary indicators based on the IB theory. Build a neural network *P*(*T*|*X*^*s*^; *θ*), where *θ* is the parameter and *T* is the latent representation of *X*^*s*^. Our goal is to learn a *T* that is maximally informative about the target *Y*, measured by the mutual information between our latent representation and the target *I*(*T*, *Y*; *θ*), where

(2) $$\begin{array}{c} I\left(T,Y;\theta \right)=\int \text{d}y\text{d}tp\left(t,y\left|\theta \right.\right)\log\frac{p\left(t,y\left|\theta \right.\right)}{p\left(t\left|\theta \right.\right)p\left(y\left|\theta \right.\right)}. \end{array}$$

To limit the dimension of *T*, add the constraint *I*(*X*^*s*^, *T*) ≤ *I*_*c*_, where *I*_*c*_ is the information constraint. This suggests the objective max_*θ*_*I*(*T*, *Y*; *θ*) s.t.*I*(*X*^*s*^, *T*; *θ*) ≤ *I*_*c*_. Equivalently, with the introduction of a Lagrange multiplier *β*, we can maximize the objective function

(3) $$\begin{array}{c} L_{\text{IB}}\left(\theta \right)=I\left(T,Y;\theta \right)-\beta I\left(X^{s},T;\theta \right), \end{array}$$

where *β* ≤ 0 controls the tradeoff. Since the main goal of the encoder is to reduce the overfitting, *β* is set at a relatively large number. The parameter sensitivity experiment in Section 4 proves the effectiveness of the module.

Then, we train a decision tree $f:x⟶\hat{y}$ and $\hat{y}\in {\left[0,1\right]}^{1\times K}$. Given a sample *x*_*i*_, the tree outputs a *K*-dimensional vector ${\hat{y}}_{i}$ and its ${\hat{y}}_{ik}$ is the *k*-th component. The probability that *x*_*i*_ belongs to class *k* is

(4) $$\begin{array}{c} p_{k}\left(x_{i}\right)=\frac{\exp\left({\hat{y}}_{ik}\right)}{\sum_{k=1}^{K}\exp\left({\hat{y}}_{ik}\right)}. \end{array}$$

For sample *x*_*i*_, the logarithm likelihood function is used to calculate the loss:

(5) $$\begin{array}{c} L\left(y_{i},f\left(x_{i}\right)\right)=-\overset{K}{\underset{k=1}{\sum }}y_{ik}\log\;p_{k}\left(x_{i}\right). \end{array}$$

To minimize the residual error of the first tree, we use the negative gradient of the loss function as the target value of the second round of training. Corresponding to the target *y* of the first round of training, the target value of the second round of training is calculated as follows:

(6) $$\begin{array}{c} r_{ik}=\frac{\partial L\left(y_{i},f\left(x_{i}\right)\right)}{\partial f\left(x_{i}\right)}=y_{ik}-p_{k}\left(x_{i}\right), \end{array}$$

where *r*_*ik*_ is the negative gradient error of class *k* corresponding to sample *x*_*i*_.

Taking *r*_*ik*_ as the target and *T* as the input data, train the second tree. Record the set of all leaf nodes of the second tree as *R*. Each leaf node has a *K*-dimensional vector as the output. The goal of the second round of training is to minimize the loss between *f*(*x*_*i*_) and *y*_*i*_. The best value for the *k*-th component in *j*-th leaf node *R*_*j*_ therefore is:

(7) $$\begin{array}{c} c_{jk}=\arg\;\underset{c_{jk}}{\min}\overset{N}{\underset{i=1}{\sum }}\overset{K}{\underset{k=1}{\sum }}L\left(y_{ik},{\hat{y}}_{ik}+\overset{J}{\underset{j=1}{\sum }}c_{jk}I\left(x_{i}\in R_{j}\right)\right), \end{array}$$

where *N* is the number of training samples and *I* is an indicator function; that is, *I*(*x*_*i*_ ∈ *R*_*j*_) = 1 if *x*_*i*_ ∈ *R*_*j*_, and 0 otherwise.

However, the above formula is difficult to obtain the optimal solution, so the approximate optimal solution is used instead:

(8) $$\begin{array}{c} c_{jk}=\frac{K-1}{K}\frac{\sum_{x_{i}\in R_{j}}r_{ik}}{\sum_{x_{i}\in R_{j}}\left|r_{ik}\right|\left(1-\left|r_{ik}\right|\right)}. \end{array}$$

For preventing overfitting and improving the diversity of transformed features, only a part of the original features is randomly resampled in the training process of the second decision tree. The process is repeated many times, just similar to the generation process of a random forest. If the selection ratio is *η* and the repetition number is *h*, the probability that each original feature has been selected at least once is 1 − (1 − *η*)^*h*^.

In the experiment, the value of *η* is set 0.5, and the value of *h* is set 200. When *K* = 3, each sample generates a 3-dimensional feature. Therefore, each original sample *x* is transformed into a 600-dimensional feature *z*_2_ by our proposed method.

Finally, the input feature *Z* of the cascaded forest is obtained by splicing *Z*_1_, *Z*_2_, and *X*.

### 3.3. Pruning Cascade Forest

**Figure 3:**
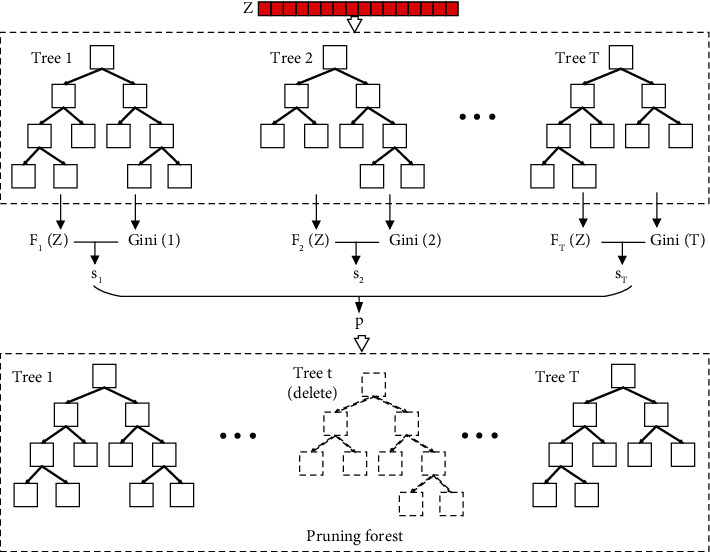
Pruning cascade forest. According to the classification results and Gini value of leaf nodes, each tree in the forest was evaluated and formed a probability vector. Pruning the forest according to the vector.

In the cascade forest structure, the output of each layer is obtained by averaging the outputs of different forests. The output of each forest is obtained by averaging the output of all decision trees. However, many features of data records in the data set for predicting overall survival were collected from the postoperative follow-up visit of the patients. Therefore, the data set has many missing values and some wrong values. As a result, it is difficult for the training model to converge rapidly or even unable to converge. Considering the characteristics of the data set, we thus propose a pruning-based cascade forest, as shown in Figure 3.

Given an instance, each forest will produce an estimate of the class distribution by counting the percentage of different classes of training examples at the leaf node where the concerned instance falls. That is, *F* : *z*⟶[0, 1]^*Tr*×*K*^, where *Tr* is the number of decision trees in the forest and *K* is the number of classifications.

A scoring index *s*_*t*_ is designed to evaluate the decision trees in forest *F*.

(9) $$\begin{array}{c} s_{t}=\frac{1}{n}\overset{n}{\underset{i=1}{\sum }}F_{t}\left(z_{i}\right)\hat{y_{i}}+\alpha {\text{Gini}}_{t}. \end{array}$$

Gini_*t*_ is the final Gini value of the *t*-th decision tree, which is used to measure the purity of the leaf nodes. And *α* is a parameter used to adjust the proportion. *s*_*t*_ is a *Tr*-dimensional score vector, which is used to measure the performance of each tree in a forest. If the decision tree with a lower score is deleted directly, it is easy to be overfitting. Therefore, a probability vector *p*_*t*_ is calculated based on the score vector *s*_*t*_. For each decision tree *t*, it is preserved according to the probability *p*_*t*_.

(10) $$\begin{array}{c} p_{t}=\frac{\exp\left(s_{t}\right)}{\underset{t\in Tr}{\max}\left\{\exp\left(s_{t}\right)\right\}}. \end{array}$$

The probability is normalized to make sure that the forest does not become an empty collection.

### 3.4. Prediction Procedure and Hyperparameters

Given a test instance, it will go through the multiview feature extraction module to produce its corresponding transformed feature representation and then follow the cascade until the last level of the tree. The final prediction will be obtained by aggregating the four 3-dimensional class vectors at the last level and taking the class with the maximum aggregated value.

Table 1 summarizes the hyperparameters of MVDF, where the values used in our experiments are given.

## 4. Experiments

In this section, we conduct several experiments for answering the following two questions: firstly, whether the deep forest can contribute to the accuracy improvement for overall survival (OS), and secondly, whether the improvement strategies proposed can improve the classification results. In the following, we describe the preprocessing of data, experiment settings, and report the evaluation results of experiments.

### 4.1. Data Sets and Preprocessing

Our experiments are conducted on GCOS (gastric cancer overall survival). The real-world data set consists of 939 patients with gastric cancer who underwent D2 gastrectomy between December 2008 and June 2013 at West China Hospital of Sichuan University. The patients from the data set have a mean age of 57.8 (standard deviation = 11.68, ranging from 24 to 86) years. Other clinicopathologic variables in the study group are reported in Table 2. Each record has 61 column indicators, with being labeled as less than 3-year OS, 3-year to 5-year OS, and more than 5-year OS. The distribution of categories is described in Table 3. Therefore, OS prediction in this study is equivalent to a three-classification task.

Due to the incompleteness of the medical data, we employ different ways to fill the missing values. For example, we use the respective average value to fill the missing height and weight by sex and a unified value to fill the missing physiological indicators including hemoglobin and neutrophil ratio before surgery, as well as the month after the operation. Moreover, the large ranges of values of the indicators have been normalized. After preprocessing the data, we randomly select thirty percent of the records in each class as the testing set, with the specific numbers listed in Table 3.

We also conduct experiments on public data sets of PSP (patient survival prediction) [39] and PST (patient survival after one year of treatment) [40]. In particular, PSP consists of 91713 records with 85 indicators. After removing the patient-ID, hospital-ID, and encounter-ID, the remaining indicators are divided into four views: basic information of patient, disease, treatment methods, and pathological indexes. PST consists of 23098 records with 16 indicators. Since some indicators have no names and descriptions, they are not divided into different views. Both of them are binary classification data sets. A label of 0 indicates that the patient is alive, while a label of 1 implies death. The sample distributions are given in Table 3.

### 4.2. Compared Methods

To demonstrate the performance of the pruning-based resampling deep forest, we compare the proposed method with the following classification approaches:

- Random forest (RF) [41]: in this method, we choose Gini values to partition the feature space and build a great number of decision trees. We refer to a prognostic value prediction method in cancer which is already validated
- Support vector machine (SVM) [42]: SVM is a widely used baseline method for classification. In this work, the SVM classifiers are trained with LibSVM which is widely used for the classification task of medical data
- Multimodal hypergraph (MMHG) [43]: MMHG is a multimodal hypergraph classification method proposed in 2017 for the postoperative prediction problem. Compared with common machine learning methods, this method achieves better classification results in the field of overall survival prediction after D2 gastrectomy for gastric cancer
- Multigrained cascade forest (gcForest) [15]: gcForest is a deep forest model which has achieved good results in many fields. To control variables for comparison, the hyperparameter values in gcForest are set as the same as those in our approach
- Multiview deep forest (MVDF): we construct the deep forest model based on the characteristics of clinical data sets and professional knowledge. The model structure and hyperparameters are detailed in the previous sections

The data set for cancer OS prediction is too small to train a deep neural network. As such, we do not compare our method with the deep learning model other than gcForest.

### 4.3. Results and Discussion

First, we will report the classification performances of the proposed MVDF compared with the baselines in Table 4 against the three databases. The performance is measured by accuracy that is the number of samples correctly predicted divided by the total number of test samples. In detail, the classification results produced by RF and SVM are similar, while the multimodal hypergraph algorithm performs better because it considers the correlation between different kinds of data. The improvements in constructing deep forests are quite prominent. To be specific, the hypergraph-based classifier outperforms the baseline by approximately 8% on the average accuracy. With respect to deep forest models, they achieve at least 3% of the average improvement over the hypergraph. After the introduction of multiview feature learning method and pruning strategy, the accuracy of the MVDF model is improved by 4% compared with gcForest. Thus, we may draw such a conclusion that the deep forest model combining information compression can contribute to overall survival classification.

PSP and PST are binary classification data sets. The confusion matrix of classification results is shown in Table 5. Compared with SVM and RF, the deep model has no significant improvement in accuracy. This is because the PSP data set is indeed imbalanced, in which the positive samples account for less than 10%. In addition, the dimension of PST data records is small, which makes it difficult to take advantage of the depth model in mining nonlinear relationships. On the other hand, the deep forest methods have some advantages in recall and F1 values. This demonstrates that this integrated approach can alleviate the impact of data imbalance to some extent. Especially in the clinic, the misjudgment of true samples (unable to survive) may affect the diagnosis and treatment methods, which need to be avoided. The above experiment shows that MVDF also has a positive effect on the survival prediction of common diseases other than cancer.

**Figure 4:**
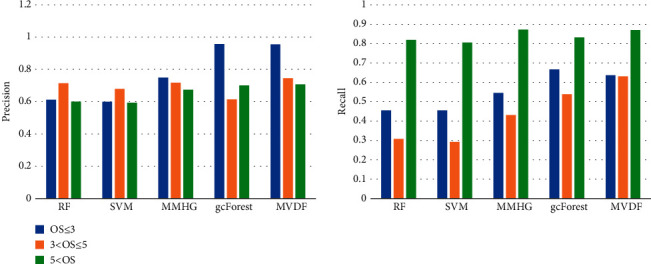
Performance under precision and recall comparisons of different approaches on three classes.

For better comparison, Figure 4 shows the prediction results of the five methods for each class in GCOS. A significant experimental result is that the recall for OS ≥ 5 years is much higher than that for the other two categories. In contrast, the corresponding precision is lower. The possible reasons are as follows:

- There are many reasons and omens for patients' short survival, and the collected indicators do not cover all related factors. This may be one reason for the low recall. At the same time, some standards of short overall survival are significant, such as large age and multiple metastasis, leading to high precision
- The imbalance of the data distribution of the data set leads to the categorical inclination. More long-term survival training samples make it easier for MVDF to predict that the patient can survive more than 5 years
- The generalization ability of the model can be improved through feature compression and resampling. Compared with gcForest, the recall of the two categories with fewer samples is significantly improved

### 4.4. Ablation Experiments and Parameter Analysis

To verify the effectiveness of the modification, we conduct ablation experiments, with the results shown in Table 6. For each class, it can be regarded as a binary classification task. Therefore, in addition to the accuracy, we add recall, precision, and F1 values as the evaluation metrics. It can be seen from the results that the two feature extraction methods are of great help to improve the recall, especially for the two classifications with fewer samples. However, it is not dominant in the comparison of a single category. The experimental results show that the classification boundary is softer, rather than relying on one or several initial metrics. This demonstrates the advantages of fusion multiview data. The pruning strategy slightly reduces the prediction accuracy; this is because our pruning strategy reduces the diversity of deep forests and is more prone to overfitting. Therefore, this strategy needs to be used together with more diverse features. Overall, the performance of our model gcForest is significantly improved over the compared models.

**Figure 5:**
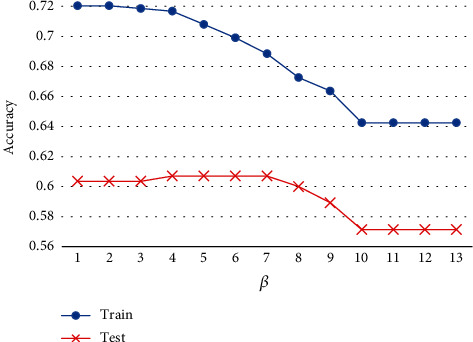
Sensitivity analysis of *β* in the function of Equation (3).

We compare the features learned from training data and test data to verify the effect of the IB theory in MVDF. Because the features are also represented as classification vectors, the accuracy of feature *z* can be used as an evaluation indicator. As shown in Figure 5, when *β* = 0, there is no restriction on the mutual information between *Z* and *X*. In this case, the result is equal to input all secondary indicators. With the increase of *β*, the accuracy of training data declines while the accuracy of test data changes little. It shows that feature compression based on the IB theory can eliminate redundant information and reduce the overfitting of the model.

**Figure 6:**
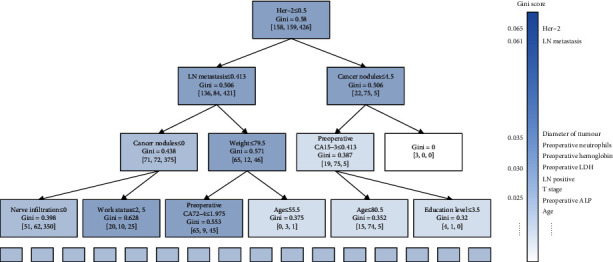
A decision tree in MVDF and the important indicators selected by forests and doctors.

To show the interpretability of the tree model, Figure 6 shows a decision tree in some deep forests. The parameters in each node include the division standard, Gini value, and training sample distribution of the node. Due to the interpretability of parameters, each decision tree can be regarded as a clinical decision path. Take the decision tree in the figure as an example. Firstly, check the HER-2 value of the patient. Suppose HER‐2 = 2 > 0.5, continue to select indicators according to the path on the right. Then, if the cancer nodules more than 4.5, the case falls in a leaf node with tree training samples of OS ≤ 3 years. It simulates the diagnosis process of a doctor. The deep forest is equivalent to the integration of multiple decision paths, which simulates the consultation of doctors. In addition, four of the five important indicators given by doctors, T stage, number of metastatic local lymph nodes (LN metastatic), lymph node positive rate, and diameter of tumour, are included in the top ten important indicators selected by random forests. It also reflects the interpretability advantage of the tree structure.

**Figure 7:**
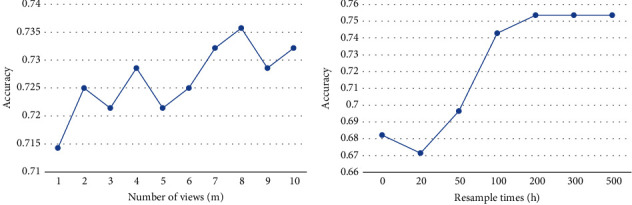
Sensitivity analysis of the parameters *m* and *h* in MVDF.

In addition, we carried out further experiments on parameter sensitivity analysis, especially for *m* (number of views) and *h* (resampling times). The experimental results are shown in Figure 7. As mentioned before, different types of detection indicators in this study are regarded as *m* views, and the features within and between views are studied, respectively. To distinguish the effectiveness of data division and view division, the data are divided randomly into 1-10 categories as control experiments. The results are shown in Figure 7. It can be seen that data division is not helpful to distinguish better features, as the data in each view is correlated. In contrast, our MVDF model is effective in capturing such correlations.

Finally, Figure 7 also shows the sensitivity of MVDF with respect to parameter *h*. If *h* is set 0, it means that the prediction is based only on the important indicators selected by doctors. If a small number of secondary indicators are randomly added, the accuracy will even be reduced. With the increase in resampling times, secondary indicators, however, will also play a stable and positive role in improving the performance of MVDF.

## 5. Conclusion

In this study, we proposed a multiview deep forest model for overall survival prediction in clinic. In our proposed approach, the information bottleneck theory is introduced to reduce the length of input data, so as to avoid overfitting. The addition of pruning strategy in cascade forests also helps to improve the performance of the model. Experimental results on actual clinical data have demonstrated that the MVDF can significantly improve the classification accuracy of overall survival prediction. For future work, we will take advantage of the interpretability of deep forest and analyze the parameters combined with theoretical medical knowledge and clinical practice.

## Figures and Tables

**Table 1:** Summary of hyperparameters and default settings.

Component	Parameter	Value
Feature extraction	Number of forests	2
	Number of trees in each forest	300
	Views of original data (*m*)	5
	Resampling times (*h*)	200
	*β*	0.1
	|*z*|	10

Cascade	Number of forests	4

Forest	Number of trees in each forest	300

**Table 2:** Clinicopathologic variables of patients.

Indicator	Value
Gender	
Male	657 (70%)
Mean weight (kg)	62.67
Mean height (cm)	163.48
Female	282 (30%)
Mean weight (kg)	55.18
Mean height (cm)	152.95
Age, mean (SD) (yr)	57.8 (11.68)
Primary tumor site	
Proximal gastric	244 (25.99%)
Gastric body	208 (22.15%)
Distal gastric	476 (50.69%)
Residual gastric	11 (11.8%)
T stage	
T1	56 (5.96%)
T2	163 (17.36%)
T3	344 (36.63%)
T4	376 (40.04%)
N stage	
N0	185 (19.70%)
N1	163 224 (23.86%)
N2	199 (21.19%)
N3	331 (35.25%)
Lauren histotype	
Intestinal	276 (29.39%)
Diffuse	506 (53.89%)
Mixed	157 (16.72%)
Tumor size, mean (SD) (cm)	5.198 (2.587)
Postoperative chemotherapy	555 (59.11%)
Postoperative adjuvant radiation	151 (16.08%)

Data are the number of patients and percentage (%).

**Table 3:** Distributions of the samples. For GCOS data set, the samples are divided into three categories based on the survival of 3 and 5 years after surgery. For PSP and PST, label 0 indicates that the patient is alive, while label 1 implies death.

GCOS			PSP		PST	
<3 years	3-5 years	>5 years	1	0	1	0
223	219	497	7915	83798	8495	14603

**Table 4:** Classification accuracy of different approaches on the three data sets GCOS, PSP, and PST.

Method	GCOS	PSP	PST
Random forest	61.43%	91.78%	94.33%
Support vector machine	60.36%	91.78%	94.20%
Multimodal hypergraph	69.28%	91.83%	94.35%
gcForest	72.38%	92.00%	94.65%
MVDF	75.42%	92.39%	94.61%

**Table 5:** Classification results of different approaches on the two data sets PSP and PST. They are compared under accuracy, precision, recall, and F1.

Method	PSP				PST			
	Accuracy	Precision	Recall	F1	Accuracy	Precision	Recall	F1
RF	91.78%	60.53%	14.74%	23.71%	94.33%	94.55%	45.41%	61.36%
SVM	91.78%	64.29%	16.88%	26.73%	94.20%	94.22%	45.40%	61.27%
MMH	91.83%	62.79%	16.88%	26.60%	94.35%	95.22%	46.70%	62.66%
gcForest	92.00%	63.64%	17.95%	28.00%	94.65%	96.92%	47.83%	64.05%
MVDF	92.39%	74.36%	18.59%	29.74%	94.61%	96.86%	47.16%	63.44%

**Table 6:** Ablation experiment on GCOS data set under recall, precision, F1, and accuracy. On the basis of gcForest, two feature extraction methods and on pruning method are compared.

Method	Classification	Recall	Precision	F1	Accuracy
gcForest	OS ≤ 3 years	66.67%	95.65%	78.57%	
	3 ≤ OS ≤ 5 years	53.85%	61.40%	57.38%	72.5%
	5 years ≤ OS	83.22%	70.06%	76.07%	

Multiview feature extraction	OS ≤ 3 years	65.15%	84.31%	73.50%	
	3 ≤ OS ≤ 5 years	58.46%	79.17%	67.26%	74.64%
	5 years ≤ OS	85.91%	70.72%	77.58%	

IB theory-based feature extraction	OS ≤ 3 years	60.61%	76.92%	67.80%	
	3 ≤ OS ≤ 5 years	53.85%	76.09%	63.06%	73.57%
	5 years ≤ OS	87.92%	71.98%	79.15%	

Pruning cascade forest	OS ≤ 3 years	54.55%	94.74%	69.23%	
	3 ≤ OS ≤ 5 years	66.15%	58.90%	62.32%	71.43%
	5 years ≤ OS	81.21%	71.60%	76.10%	

MVDF	OS ≤ 3 years	63.64%	95.45%	76.36%	
	3 ≤ OS ≤ 5 years	63.08%	74.55%	68.33%	75.36%
	5 years ≤ OS	85.91%	70.72%	77.58%	

## Data Availability

Previously reported (patient survival prediction) data were used to support this study and are available at 10.34740/KAGGLE/DSV/2972359. These prior studies (and data sets) are cited at relevant places within the text as references. Previously reported (patient survival after one year of treatment) data were used to support this study and are available at 10.34740/KAGGLE/DSV/1546097. These prior studies (and data sets) are cited at relevant places within the text as references. The gastric cancer overall survival data used to support the findings of this study were supplied by Zedong Du and Qiu Li under license and so cannot be made freely available. Requests for access to these data should be made to Zedong Du (amos1985@163.com).
